# Polymorphisms of nucleotide factor of activated T cells cytoplasmic 2 and 4 and the risk of acute rejection following kidney transplantation

**DOI:** 10.1007/s00345-017-2117-2

**Published:** 2017-11-04

**Authors:** Zijie Wang, Haiwei Yang, Shuhui Si, Zhijian Han, Jun Tao, Hao Chen, Yuqiu Ge, Miao Guo, Ke Wang, Ruoyun Tan, Ji-Fu Wei, Min Gu

**Affiliations:** 1 Department of Urology, The First Affiliated Hospital with Nanjing Medical University, Nanjing, 210029 People’s Republic of China; 2 Research Division of Clinical Pharmacology, The First Affiliated Hospital with Nanjing Medical University, Nanjing, 210029 People’s Republic of China; 3 School of Public Health, Nanjing Medical University, Nanjing, 211166 People’s Republic of China

**Keywords:** Kidney transplantation, Nuclear factor of activated T cells C2, Nuclear factor of activated T cells C4, Single nucleotide polymorphism, Next-generation sequencing

## Abstract

**Background:**

Acute rejection (AR) is a common complication of kidney transplantation. Nuclear factors of activated T cells (NFATs) are transcription factors involved in the activation of T lymphocytes, but their association with AR is unclear.

**Methods:**

This retrospective, case–control study included 200 renal transplant recipients who were divided into the AR group (*n* = 69) and stable group (*n* = 131). Their blood samples were collected, and DNA was extracted from the whole blood. High-throughput next-generation sequencing was used to identify single nucleotide polymorphisms (SNPs) of the *NFATC2* and *NFATC4* genes. The correlation of these SNPs with AR was determined by logistic analysis.

**Results:**

Seventy-one SNPs of the *NFATC2* and *NFATC4* genes were identified by the sequencing and Hardy–Weinberg equilibrium analyses. After adjusting for age, gender and immunosuppressive protocols, 27 SNPs were correlated with AR, of which the SNP rs2426295 of the *NFATC2* gene showed a significant correlation with AR in the HET model (AA vs. AC: OR = 0.43, 95% CI = 0.19–0.98, *P* = 0.045), but no significant *NFATC4* SNPs were identified.

**Conclusions:**

Our study shows that the rs2426295 variant of the *NFATC2* gene is significantly associated with the occurrence of AR following kidney transplantation. And patients with AA genotypes in rs2426295 are inclined to suffer from AR pathogenesis.

**Electronic supplementary material:**

The online version of this article (10.1007/s00345-017-2117-2) contains supplementary material, which is available to authorized users.

## Introduction

Kidney transplantation is the optimal and most cost-effective method of renal replacement therapy for patients with end-stage renal diseases [1, 2]. However, its wide applicability is limited by various complications, such as acute rejection (AR), chronic allograft dysfunction and immunosuppressive agent-related nephrotoxicity. Among these complications, AR is an important cause of graft loss that may occur at any time point in the lifetime of renal transplant recipients, independent of age and gender [3]. Therefore, understanding the mechanisms underlying AR is of great benefit with regard to promoting short-term and long-term allograft survival [4].

Nuclear factor of activated T cells (NFAT) is the name given to a family of proteins that are expressed in most immune cells [5, 6]. The NFAT family of transcription factors is composed of five members—Nfatc1–4 and Nfat5—which integrate calcium signals to modulate gene expression and contribute to the growth, differential and immune responses to endogenous and extrinsic stimulations [7, 8]. The classical members of the gene family are *NFAT1* (also known as *NFATp* or *NFATc2*), *NFAT2* (*NFATc* or *NFATc1*), *NFAT3 (NFATc4)* and *NFAT4* (*NFATx* or *NFATc3*) [9–13]. The canonical NFAT pathway is calcium dependent, and upon its activation, NFATs are dephosphorylated by the phosphatase calcineurin, which is also calcium dependent [14]. Specifically, calcineurin modulates the translocation of NFAT proteins from the cytoplasm to the nucleus of activated cells by interacting with an N-terminal regulatory domain that is conserved in the NFAT family [15–17]. Classic immunosuppressive agents, such as cyclosporine A (CsA) and tacrolimus, inhibit the action of calcineurin and thereby prevent the de-phosphorylation of NFATs and their subsequent nuclear translocation [18, 19].

Studies have shown that *NFATC2* mRNA is expressed mainly in peripheral lymphoid tissues such as the spleen and peripheral blood lymphocytes, and that *NFATC4* mRNA is expressed at high levels in the thymus; these findings indicate the crucial role of *NFATC2* and *NFATC4* in T-cell development [13, 20]. The *NFATC2* gene encodes a cytoplasmic component, Nfatc2, which is dephosphorylated in response to T-cell receptors and then translocated into the nucleus, where it plays a role in the modulation of gene transcripts [21]. Recent studies show that NFATC2 has a great impact on the development and function of regulatory T cells, and that it positively or negatively modulates the immune response depending on the antigen present [22, 23]. Moreover, NFATC2 has been found to play a subtle and selective role in maintaining a state of anergy for B-cell receptor stimulation by repressing the transcription of other NFAT family members, such as NFATc1 and NFATc3 [24]. In addition, *NFATC2* mRNA was found to be upregulated in activated T cells and NK cells through a CsA-dependent mechanism [10]. However, no related study was designed to explore the relationship between the *NFATC2/4* and acute rejection following kidney transplantation. Based on all these findings, we firstly hypothesized that the *NFATC2* and *NFATC4* genes play a role in the activation of T cells during AR episodes following kidney transplantation.

In this study, for the first time we performed a comprehensive analysis of single nucleotide polymorphisms (SNPs) of *NFATC2/C4* in renal transplant recipients by using next-generation sequencing (NGS) to investigate the potential association between *NFATC2/C4* SNPs and AR pathogenesis following kidney transplantation.

## Methods

### Ethics statement

The study protocol was in accordance with the ethical standards of the Declarations of Helsinki and Istanbul. The procedures were limited to the living-related transplantation of kidney tissues to lineal or collateral relatives not beyond the third degree of kinship or transplantation of kidney tissues from cadaveric allograft donors after cardiac death. The protocol of this study was approved by the local ethics committee of the First Affiliated Hospital with Nanjing Medical University. Written informed consent was obtained from all the transplant recipients. Moreover, none of the transplant donors were from a vulnerable population, and their written informed consent was obtained.

### Study design

This was a retrospective, single-center, case–control study. A total of 200 renal transplant recipients who underwent kidney transplantation between 1st February 2010 and 1st December 2015, at our center of the First Affiliated Hospital with Nanjing Medical University, were enrolled in this study. According to the study criteria, we included (1) patients aged more than 18 years or less than 60 years; (2) patients who had experienced at least one AR episode after kidney transplantation that was confirmed by histological examination with hematoxylin–eosin staining and immunohistological staining according to the Banff07 criteria [25] (these patients were assigned to the AR group); (3) patients with stable serum creatinine levels (< 120 μmol/L; fluctuation, < 20%) for at least 3 months who had not experienced any AR episodes, delayed graft dysfunction or opportunistic infection after kidney transplantation (these patients were assigned to the stable group); and (4) patients who had undergone post-transplant follow-up for more than 6 months. Further, we excluded (1) patients who did not fulfill the inclusion criteria, (2) patients with chronic viral infections such as HIV and chronic viral hepatitis B and C, and (3) pregnant women. The medical records of the enrolled patients were critically reviewed, and relevant data were extracted independently by two authors (Z.J. Wang and K. Wang).

### Immunosuppressive protocols

All recipients received immunosuppressive protocols that included three or four drugs: cyclosporin A (CsA, *n* = 98) or tacrolimus (TAC, *n* = 102) combined with mycophenolate mofetil (MMF) and prednisone, with or without sirolimus (*n* = 15) (Table 1). Methylprednisolone was intravenously administered as a dosage of 500 mg/day on the day of surgery and until 2 days after the kidney transplantation. Following this, the dosage was reduced to 400, 300, 200 mg and then 80 mg on each subsequent day. This was followed by oral administration of prednisone at 30 mg/day as maintenance therapy. Moreover, 20 mg basiliximab was intravenously administered at 30 min before the surgery and on the fourth post-transplant day. MMF was administered at 24 to 48 h after transplantation, and the starting dosage was 0.75 g/d to 1.0 g/d (BID). The dose was adjusted according to the serum creatinine (Scr) and drug concentrations. The starting dosage of CsA and TAC was 8 mg/kg/day and 0.2 mg/kg/day, respectively; these dosages were later adjusted according to the Scr levels and drug concentrations. In the case of patients who experienced AR episodes, methylprednisolone was intravenously administered at a dosage of 200 mg/day for 3–5 days.

**Table 1:** Comparison of baseline characteristics between AR and stable subjects

Characteristics	Stable group	AR group	*P* value
Case number	131	69	NS
Age (years, mean ± SD)	41.29 ± 1.98	40.88 ± 2.97	NS
Male (%)	62.60	60.86	NS
PRA (%) before renal transplant	0.00	0.00	–
Immunosuppressive protocol			NS
Pred+ MMF+ CsA	64	26	
Pred+ MMF+ TAC	59	36	
Pred+ MMF+ CsA+ SIR	4	4	
Pred+ MMF+ TAC+ SIR	4	3	

*AR* acute rejection; *NS* not significant; *SD* standard deviation; *HLA* human lymphocyte antigen; *PRA* panel reactive antibody; *Pred* prednisone; *MMF* mycophenolate mofetil; *CsA* cyclosporin A; *TAC* tacrolimus; *SIR* sirolimus

### Sample collection, preparation and NGS

Peripheral blood samples (2 ml) from each recipient were collected. In particular, blood samples from recipients in the AR group were taken before the intravenous administration of methylprednisolone and the allograft biopsy. The blood samples were transferred to the laboratory and stored in the refrigerator at − 80°C.

DNA of the blood samples was extracted using the QIAmp DNA mini kit according to the manufacturer’s instructions (Qiagen, Hilden, Germany). The concentration and purity of genomic DNA (gDNA) were quantitatively analyzed using NanoDrop ND2000 (Thermo, MA, USA), while gene integrity was assessed using agarose gel electrophoresis. The gDNA samples extracted were considered to be acceptable if the total mass was ≥ 1 μg, and the A260/A280 absorbance ratio was ≥ 1.80 and ≤ 2.0. The gDNA was added to a mixture of upstream and downstream oligonucleotides specific to the target regions of interest for hybridization (see supplementary files). Then, the gDNA was fragmented using a Bioruptor Interrupt (Diagenode, Belgium), and quantitative detection was performed to ensure that the average fragment size was 150–250 bp. Fragmentation was followed by end repair, dA tailing, and sequencing adaptor ligation with the ABI 9700 PCR instrument (ABI, USA). The adapter-ligated DNA was amplified by selective, limited-cycle PCR for five cycles and then quantitatively analyzed using the Qubit dsDNA HS assay kit (Invitrogen, USA). The prepared library (750 ng) was hybridized by overnight incubation (for 8–16 h) at 65°C with 11 µl of hybridization blocking buffer (Allwegene, China), 20 µl of hybridization buffer (Allwegene, China), and a mixture of 5 µl RNase block (Invitrogen, USA) and 2 µl probes (Allwegene, China). The hybridized products were mixed with 200 µl Dynabeads MyOne Streptavidin T1 magnetic beads (Invitrogen, USA) for 30 min at room temperature. The products were then washed two times with a wash buffer (Allwegene, China), and the mixture was amplified for 16 PCR cycles and quantitatively assessed using the Qubit dsDNA HS assay kit (Invitrogen, USA). The captured libraries were denatured and loaded onto an Illumina cBot instrument at a concentration of 12–16 pmol/l for cluster generation according to the manufacturer’s instructions. Up to 20 WUCaMP libraries were sequenced per HiSeq lane. A PhiX control (Illumina) was added to lane 8 of each flow cell.

### Analysis of NGS data

Sequencing data, such as the number of altered chromosomes, genomic alterations, and the depth of the sequencing coverage, were analyzed. All analyses were based on the human reference sequence UCSC build hg19 (NCBI build 37.2) using the Burrows–Wheeler Aligner. Local alignment and removal of duplicates were performed using the Genome Analysis Toolkit (Version 3.7-0, Broad Institute, Cambridge, MA, USA) and the Picard software (Broad Institute, Cambridge, MA, USA). Detection of SNPs was performed using dbSNP 132. Damaging or deleterious SNPs were predicted using the Gemini software, and prediction tools, including sorting intolerant from tolerant and polymorphism phenotyping, were used for analysis of all human non-synonymous SNPs. In addition, putative somatic variant calls were detected using two separate programs, MuTect 1.1.5 and VarScan 2.3.6, by pairing each sample with its matched blood sample.

### Statistical analysis

Conformance to the Hardy–Weinberg equilibrium (HWE) was determined based on gene frequencies obtained from a single gene counting. The Chi-square test was used to compare the observed and expected values. To perform genotype association analysis, five models were used: the dominant model (minor allele homozygotes plus heterozygotes vs. major allele homozygotes), recessive model (minor allele homozygotes vs. heterozygotes plus major homozygotes), additive model (major homozygotes vs. heterozygotes vs. minor homozygotes), HET model (major homozygotes vs. heterozygotes) and HOM model (major homozygotes vs. minor homozygotes). Genotypic frequencies of the control and AR groups were compared by the Chi-square test. In addition, we explored linkage disequilibrium blocks using Haploview version 4.2. Odds ratios (ORs) and 95% confidence intervals (95% CIs) were calculated using the SPSS 13.0 software (SPSS Inc., Chicago, IL, USA). *P* < 0.05 was considered to indicate statistical significance. The OR provides an effect estimate: when its value is less than 1, a protective effect can be assumed, whereas when it is more than 1, the disease risk is considered to be increased. In addition, the genotypic distributions of the *NFATC2/C4* SNPs in recipients with AR and stable recipients were analyzed using logistic regression models adjusted for age, gender and immunosuppressive protocol.

## Results

### Demographic characteristics

The baseline clinical characteristics of the renal transplant recipients in our study are presented in Table 1. A total of 69 recipients who experienced at least one AR episode (42 men and 27 women) were assigned to the AR group, and 131 recipients who had not experienced any AR episodes (82 men and 49 women) were assigned to the stable group. No significant differences (*P* > 0.05) were observed in baseline characteristics, such as age, gender and immunosuppressive protocol, between the stable and AR groups. No patient in two groups was tested with positive results of panel reactive antibodies before kidney transplantation.

### Association of *NFATC2*/*NFATC4* SNPs with AR

A total of 71 SNPs of the *NFATC2* and *NFATC4* genes (*NFATC2*: 34 SNPs, *NFATC4*: 37 SNPs) were identified using the NGS technology and HWE analysis (Supplemental Tables 1 and 2). The genetic distribution of these SNPs in the AR and stable group are shown in Supplemental Tables 3 and 4. Among these SNPs, 9 *NFATC2* SNPs and 11 *NFATC4* SNPs are being reported here for the first time. We selected 27 SNPs with MAF (minor allele frequency) more than 0.05 to further test the relationship with the AR episodes.

After adjustment for age, gender and immunosuppressive protocols in all five models (additive, dominant, recessive, HOM and HET), we found that rs2426295 on the *NFATC2* gene was significantly correlated with the risk of AR episodes following kidney transplantation in the HET model (AA vs. AC: OR = 0.43, 95% CI = 0.19–0.98, *P* = 0.045, Table 2). No significant association was observed for the other 26 SNPs in all the models (Supplemental Table 5).

**Table 2:** Regression analysis for age-, gender- and immunosuppressive protocol-adjusted rs2426295 genetic polymorphisms located on *NFATC2 gene* among recipients with acute rejection

SNPs	Model	OR	95% CIs	*P* value
rs2426295				
	Additive	0.62	0.31, 1.21	0.16
	Dominant	0.49	0.23, 1.07	0.073
	Recessive	1.59	0.25, 9.99	0.62
	HET	0.43	0.19, 0.98	**0.045**
	HOM	1.35	0.21, 8.51	0.75

Bold value is statistically important

*SNPs* single nucleotide polymorphisms; *OR* odds ratio; *CIs* confidential intervals

## Discussion

In the present study, we used NGS to identify SNPs of the *NFATC2* and *NFATC4* genes that are associated with AR following renal transplant. Our findings indicated that the SNP rs2426295 on the *NFATC2* gene showed a significant correlation with the occurrence of post-transplantation AR episodes in renal transplant patients. This finding implies that recipients carrying the AC genotype may be at a higher risk of AR after kidney transplantation than those with the AA genotype; thus, the variation from A to C in the rs2426295 SNP may influence transcription of the *NFATC2* gene and positively contribute to the activation of T cells. Consistent with our findings, a genome-based trial observed that the *NFATC2* variant rs6021191 is associated with the risk of asparaginase hypersensitivity, and that inhibition of NFATC2 might mitigate sensitization to asparaginase [26]. Combined with the present findings, this provides genetic evidence for the role of NFATC2 in the regulation of T-cell activation and immune responses.

In our study, we did not observe any statistical difference in the frequency of rs10141896 or other SNPs of the NFATC4 gene between the two groups. However, there is direct evidence to show that the expression of NFATC4 mRNA is significantly increased in patients with renal allograft dysfunction caused by chronic rejection [27]. This difference could be explained by the ethnicity of the patients in both studies and the small sample size in the present study. However, one study has shown that the rs10141896 T variant on the NFATC4 gene was associated with a lower accumulative incidence of new-onset diabetes after transplantation (NODAT), and that patients carrying a tagging SNP for one of the five dominant NFATC4 haplotypes, T-T-T-T-G, had a reduced risk of NODAT [28]. Thus, there is no clear finding about the role of NFATC4 genetic polymorphisms in the regulation of immune responses in renal transplant recipients. To further investigate the role of NFATC4 in the risk of AR, a large-scale multi-center clinical trial should be designed and carried out.

Our study revealed the potentially significant effect of the NFATC2 gene on the pathogenesis of rejection following the kidney transplantation, in which the mutation of NFATC2 rs2426295 contributed to the protection of allograft. This finding could provide the novel insight to the research of rejection in vivo and in vitro. Meanwhile, we are also aware of the shortage of our study, and further well-designed study, including the large-scale confirmation of this mutation on NFATC2 gene and the functional experiments in vitro, are in progress to validate our findings in this manuscript.

In summary, in the present retrospective case–control study, we identified 71 *NFATC2/NFATC4* SNPs using the NGS technology. Moreover, rs2426295 on the *NFATC2* gene was found to be statistically correlated with the risk of AR episodes following kidney transplantation. And patients with AA genotypes in rs2426295 are inclined to suffer from AR pathogenesis. To confirm our findings, a large-scale, well-designed study should be conducted.

## Electronic supplementary material

Below is the link to the electronic supplementary material.

Supplementary material 1 (DOCX 17 kb)

Supplementary material 2 (DOCX 17 kb)

Supplementary material 3 (DOCX 28 kb)

Supplementary material 4 (DOCX 29 kb)

Supplementary material 5 (DOCX 28 kb)

## Abbreviations

AR: Acute rejection
NFATs: Nuclear factors of activated T cells
SNPs: Single nucleotide polymorphisms
NGS: Next-generation sequencing
CsA: Cyclosporin A
TAC: Tacrolimus
MMF: Mycophenolate mofetil
Scr: Serum creatinine
gDNA: Genomic DNA
HWE: Hardy–Weinberg equilibrium
ORs: Odds ratios
95% CIs: 95% confidence intervals
NODAT: New-onset diabetes after transplantation
MAF: Minor allele frequency
