# Supplementary material for: Polymorphisms of nucleotide factor of activated T cells cytoplasmic 2 and 4 and the risk of acute rejection following kidney transplantation
Source: World J Urol. 2017 Nov 4;36(1):111–6. doi: 10.1007/s00345-017-2117-2 (PMC5758697; doi:10.1007/s00345-017-2117-2)
Supplement: Supplementary file 1 — Supplementary material 1 (DOCX 17 kb) [file 345_2017_2117_MOESM1_ESM.docx]

**(Supplemental) Table 1. Single nuclear polymorphisms identified in *NFATC2* gene in our study.**

| SNPs | Locations | | function |
| --- | --- | --- | --- |
|  | chromosome | position |  |
| rs577945673 | chr20 | 50007799 | UTR3 |
| rs781068976 | chr20 | 50008010 | intronic |
| - | chr20 | 50015223 | UTR3 |
| rs564569127 | chr20 | 50015267 | exonic |
| rs2426295 | chr20 | 50015299 | intronic |
| - | chr20 | 50015331 | intronic |
| rs371799902 | chr20 | 50048522 | intronic |
| - | chr20 | 50049069 | exonic |
| - | chr20 | 50049269 | exonic |
| rs559062496 | chr20 | 50049321 | intronic |
| rs369140336 | chr20 | 50049340 | intronic |
| - | chr20 | 50049351 | intronic |
| - | chr20 | 50052127 | intronic |
| rs772713845 | chr20 | 50052243 | exonic |
| rs139882911 | chr20 | 50070985 | intronic |
| rs552141847 | chr20 | 50071183 | exonic |
| rs228840 | chr20 | 50071258 | intronic |
| rs73615391 | chr20 | 50071452 | intronic |
| rs111838448 | chr20 | 50091787 | intronic |
| rs56332276 | chr20 | 50091937 | intronic |
| rs6013193 | chr20 | 50092027 | exonic |
| rs181122021 | chr20 | 50092077 | exonic |
| rs12479626 | chr20 | 50092193 | exonic |
| rs6021231 | chr20 | 50092287 | intronic |
| rs74644406 | chr20 | 50092366 | intronic |
| - | chr20 | 50139541 | intronic |
| rs188481905 | chr20 | 50139985 | exonic |
| rs758870046 | chr20 | 50140058 | exonic |
| rs3746420 | chr20 | 50140627 | exonic |
| - | chr20 | 50158881 | intronic |
| rs2869427 | chr20 | 50178965 | intronic |
| - | chr20 | 50179204 | UTR5 |
| - | chr20 | 50179316 | UTR5 |
| rs75374025 | chr20 | 50179356 | UTR5 |
