# Supplementary material for: Polymorphisms of nucleotide factor of activated T cells cytoplasmic 2 and 4 and the risk of acute rejection following kidney transplantation
Source: World J Urol. 2017 Nov 4;36(1):111–6. doi: 10.1007/s00345-017-2117-2 (PMC5758697; doi:10.1007/s00345-017-2117-2)
Supplement: Supplementary file 2 — Supplementary material 2 (DOCX 17 kb) [file 345_2017_2117_MOESM2_ESM.docx]

**(Supplemental) Table 2. Single nuclear polymorphisms identified in *NFATC4* gene in our study.**

| SNPs | Locations | | Functions |
| --- | --- | --- | --- |
|  | Chromosome | Position |  |
| - | chr14 | 24836555 | intronic |
| rs28365903 | chr14 | 24836815 | intronic |
| - | chr14 | 24836914 | intronic |
| rs200387476 | chr14 | 24837506 | UTR5 |
| rs45565135 | chr14 | 24837668 | intronic |
| - | chr14 | 24838383 | UTR5 |
| rs537893669 | chr14 | 24838557 | exonic |
| rs1955915 | chr14 | 24838621 | intronic |
| rs55651033 | chr14 | 24838937 | exonic |
| rs2229309 | chr14 | 24839083 | exonic |
| rs2228233 | chr14 | 24839165 | exonic |
| - | chr14 | 24839572 | exonic |
| - | chr14 | 24839648 | exonic |
| rs2229310 | chr14 | 24839756 | exonic |
| rs778422832 | chr14 | 24839812 | intronic |
| rs56044944 | chr14 | 24839897 | intronic |
| - | chr14 | 24839930 | intronic |
| rs10141527 | chr14 | 24841517 | intronic |
| - | chr14 | 24841650 | exonic |
| - | chr14 | 24841698 | exonic |
| rs773652389 | chr14 | 24842421 | exonic |
| rs149390527 | chr14 | 24842498 | exonic |
| rs77893724 | chr14 | 24842563 | exonic |
| rs762460806 | chr14 | 24843537 | exonic |
| rs2295298 | chr14 | 24843620 | exonic |
| - | chr14 | 24843697 | intronic |
| rs12890614 | chr14 | 24843699 | intronic |
| rs368284613 | chr14 | 24843712 | intronic |
| rs12880769 | chr14 | 24845154 | intronic |
| rs56111443 | chr14 | 24845338 | intronic |
| rs10141896 | chr14 | 24845402 | intronic |
| - | chr14 | 24845678 | exonic |
| rs7149586 | chr14 | 24845841 | exonic |
| rs2243891 | chr14 | 24846757 | UTR3 |
| rs10362 | chr14 | 24846961 | UTR3 |
| rs56006071 | chr14 | 24847063 | UTR3 |
| - | chr14 | 24847105 | UTR3 |
