# Supplementary material for: Polymorphisms of nucleotide factor of activated T cells cytoplasmic 2 and 4 and the risk of acute rejection following kidney transplantation
Source: World J Urol. 2017 Nov 4;36(1):111–6. doi: 10.1007/s00345-017-2117-2 (PMC5758697; doi:10.1007/s00345-017-2117-2)
Supplement: Supplementary file 3 — Supplementary material 3 (DOCX 28 kb) [file 345_2017_2117_MOESM3_ESM.docx]

**(Supplemental) Table 3. Genetic distributions of NFATC2 polymorphisms between AR and stable group.**

| **Genotype** | **Chromosome** | **Position** | **Stable group (n=131)** | **AR group (n=69)** | **HWE for stable group** | |
| --- | --- | --- | --- | --- | --- | --- |
|  |  |  |  |  | Χ2 | *P* value |
| rs577945673 | Chr20 | 50007799 |  |  | <0.01 | 0.99 |
| CC |  |  | 131 | 68 |  |  |
| CT |  |  | 0 | 1 |  |  |
| rs781068976 | Chr20 | 50008010 |  |  | <0.01 | 0.99 |
| GG |  |  | 130 | 69 |  |  |
| GT |  |  | 1 | 0 |  |  |
| / | Chr20 | 50015223 |  |  | 0.01 | 0.99 |
| TT |  |  | 129 | 69 |  |  |
| TC |  |  | 2 | 0 |  |  |
| rs564569127 | Chr20 | 50015267 |  |  | <0.01 | 0.99 |
| TT |  |  | 130 | 69 |  |  |
| TG |  |  | 1 | 0 |  |  |
| rs2426295 | Chr20 | 50015299 |  |  | 1.24 | 0.54 |
| AA |  |  | 96 | 58 |  |  |
| AC |  |  | 32 | 9 |  |  |
| CC |  |  | 3 | 2 |  |  |
| / | Chr20 | 50015331 |  |  | <0.01 | 0.99 |
| CC |  |  | 131 | 68 |  |  |
| CT |  |  | 0 | 1 |  |  |
| rs371799902 | Chr20 | 50048522 |  |  | <0.01 | 0.99 |
| GG |  |  | 130 | 69 |  |  |
| GA |  |  | 1 | 0 |  |  |
| / | Chr20 | 50049069 |  |  | <0.01 | 0.99 |
| GG |  |  | 130 | 69 |  |  |
| GC |  |  | 1 | 0 |  |  |
| / | Chr20 | 50049269 |  |  | <0.01 | 0.99 |
| GG |  |  | 131 | 68 |  |  |
| GA |  |  | 0 | 1 |  |  |
| rs559062496 | Chr20 | 50049321 |  |  | <0.01 | 0.99 |
| CC |  |  | 131 | 68 |  |  |
| CT |  |  | 0 | 1 |  |  |
| rs369140336 | Chr20 | 50049340 |  |  | <0.01 | 0.99 |
| CC |  |  | 130 | 69 |  |  |
| CT |  |  | 1 | 0 |  |  |
| / | Chr20 | 50049351 |  |  | <0.01 | 0.99 |
| GG |  |  | 130 | 69 |  |  |
| GA |  |  | 1 | 0 |  |  |
| / | Chr20 | 50052127 |  |  | <0.01 | 0.99 |
| TT |  |  | 131 | 68 |  |  |
| TA |  |  | 0 | 1 |  |  |
| rs772713845 | Chr20 | 50052243 |  |  | <0.01 | 0.99 |
| GG |  |  | 130 | 69 |  |  |
| GA |  |  | 1 | 0 |  |  |
| rs139882911 | Chr20 | 50070985 |  |  | 0.26 | 0.88 |
| GG |  |  | 123 | 63 |  |  |
| GA |  |  | 8 | 6 |  |  |
| rs552141847 | Chr20 | 50071183 |  |  | <0.01 | 0.99 |
| TT |  |  | 130 | 69 |  |  |
| TG |  |  | 1 | 0 |  |  |
| rs228840 | Chr20 | 50071258 |  |  | 0.41 | 0.82 |
| AA |  |  | 7 | 4 |  |  |
| AG |  |  | 43 | 22 |  |  |
| GG |  |  | 81 | 43 |  |  |
| rs73615391 | Chr20 | 50071452 |  |  | 0.01 | 0.99 |
| GG |  |  | 130 | 68 |  |  |
| GA |  |  | 1 | 1 |  |  |
| rs111838448 | Chr20 | 50091787 |  |  | <0.01 | 0.99 |
| CC |  |  | 131 | 68 |  |  |
| CT |  |  | 0 | 1 |  |  |
| rs56332276 | Chr20 | 50091937 |  |  | 1.05 | 0.59 |
| AA |  |  | 96 | 52 |  |  |
| AG |  |  | 31 | 15 |  |  |
| GG |  |  | 4 | 2 |  |  |
| rs6013193 | Chr20 | 50092027 |  |  | 0.37 | 0.83 |
| TT |  |  | 47 | 26 |  |  |
| TG |  |  | 66 | 33 |  |  |
| GG |  |  | 18 | 10 |  |  |
| rs181122021 | Chr20 | 50092077 |  |  | <0.01 | 0.99 |
| TT |  |  | 130 | 69 |  |  |
| TA |  |  | 1 | 0 |  |  |
| rs12479626 | Chr20 | 50092193 |  |  | 1.31 | 0.52 |
| TT |  |  | 110 | 60 |  |  |
| TC |  |  | 21 | 9 |  |  |
| rs6021231 | Chr20 | 50092287 |  |  | 0.17 | 0.92 |
| TT |  |  | 45 | 25 |  |  |
| TC |  |  | 67 | 32 |  |  |
| CC |  |  | 19 | 12 |  |  |
| rs74644406 | Chr20 | 50092366 |  |  | 0.19 | 0.91 |
| GG |  |  | 125 | 63 |  |  |
| GA |  |  | 6 | 6 |  |  |
| / | Chr20 | 50139541 |  |  | <0.01 | 0.99 |
| CC |  |  | 131 | 68 |  |  |
| CT |  |  | 0 | 1 |  |  |
| rs188481905 | Chr20 | 50139985 |  |  | <0.01 | 0.99 |
| GG |  |  | 131 | 68 |  |  |
| GC |  |  | 0 | 1 |  |  |
| rs758870046 | Chr20 | 50140058 |  |  | <0.01 | 0.99 |
| GG |  |  | 131 | 68 |  |  |
| GA |  |  | 0 | 1 |  |  |
| rs3746420 | Chr20 | 50140627 |  |  | 0.89 | 0.64 |
| GG |  |  | 118 | 57 |  |  |
| GC |  |  | 13 | 12 |  |  |
| rs50158881 | Chr20 | 50158881 |  |  | <0.01 | 0.99 |
| GG |  |  | 130 | 69 |  |  |
| GC |  |  | 1 | 0 |  |  |
| rs2869427 | Chr20 | 50178965 |  |  | <0.01 | 0.99 |
| GG |  |  | 112 | 60 |  |  |
| GC |  |  | 18 | 9 |  |  |
| CC |  |  | 1 | 0 |  |  |
| / | Chr20 | 50179204 |  |  | <0.01 | 0.99 |
| GG |  |  | 131 | 68 |  |  |
| GA |  |  | 0 | 1 |  |  |
| / | Chr20 | 50179316 |  |  | <0.01 | 0.99 |
| CC |  |  | 130 | 69 |  |  |
| CG |  |  | 1 | 0 |  |  |
| rs75374025 | Chr20 | 50179356 |  |  | 3.60 | 0.17 |
| CC |  |  | 118 | 62 |  |  |
| CT |  |  | 11 | 7 |  |  |
| TT |  |  | 2 | 0 |  |  |
