# Supplementary material for: Polymorphisms of nucleotide factor of activated T cells cytoplasmic 2 and 4 and the risk of acute rejection following kidney transplantation
Source: World J Urol. 2017 Nov 4;36(1):111–6. doi: 10.1007/s00345-017-2117-2 (PMC5758697; doi:10.1007/s00345-017-2117-2)
Supplement: Supplementary file 4 — Supplementary material 4 (DOCX 29 kb) [file 345_2017_2117_MOESM4_ESM.docx]

**(Supplemental) Table 4. Genetic distributions of *NFATC4* polymorphisms between AR and stable group.**

| **Genotype** | **Chromosome** | **Position** | **Stable group (n=131)** | **AR group (n=69)** | **HWE for stable group** | |
| --- | --- | --- | --- | --- | --- | --- |
|  |  |  |  |  | Χ2 | *P* value |
| / | Chr14 | 24836555 |  |  | <0.01 | 0.99 |
| CC |  |  | 130 | 69 |  |  |
| CT |  |  | 1 | 0 |  |  |
| rs28365903 | Chr14 | 24836815 |  |  | 0.44 | 0.80 |
| CC |  |  | 91 | 48 |  |  |
| CG |  |  | 36 | 21 |  |  |
| GG |  |  | 4 | 0 |  |  |
| / | Chr14 | 24836914 |  |  | <0.01 | 0.99 |
| TT |  |  | 130 | 69 |  |  |
| TC |  |  | 1 | 0 |  |  |
| rs200387476 | Chr14 | 24837506 |  |  | 0.01 | 0.99 |
| CC |  |  | 129 | 69 |  |  |
| CG |  |  | 2 | 0 |  |  |
| rs45565135 | Chr14 | 24837668 |  |  | 0.05 | 0.97 |
| GG |  |  | 91 | 48 |  |  |
| GC |  |  | 35 | 21 |  |  |
| CC |  |  | 5 | 0 |  |  |
| / | Chr14 | 24838383 |  |  | <0.01 | 0.99 |
| CC |  |  | 130 | 69 |  |  |
| CT |  |  | 1 | 0 |  |  |
| rs537893669 | Chr14 | 24838557 |  |  | 0.01 | 0.99 |
| TT |  |  | 130 | 68 |  |  |
| TC |  |  | 1 | 1 |  |  |
| rs1955915 | Chr14 | 24838621 |  |  | 2.63 | 0.27 |
| CC |  |  | 13 | 9 |  |  |
| CT |  |  | 47 | 25 |  |  |
| TT |  |  | 71 | 35 |  |  |
| rs55651033 | Chr14 | 24838937 |  |  | 0.23 | 0.89 |
| CC |  |  | 121 | 66 |  |  |
| CT |  |  | 10 | 3 |  |  |
| rs2229309 | Chr14 | 24839083 |  |  | 5.74 | 0.057 |
| GG |  |  | 102 | 50 |  |  |
| GC |  |  | 25 | 15 |  |  |
| CC |  |  | 4 | 4 |  |  |
| rs2228233 | Chr14 | 24839165 |  |  | 0.93 | 0.63 |
| CC |  |  | 40 | 18 |  |  |
| CT |  |  | 55 | 38 |  |  |
| TT |  |  | 36 | 13 |  |  |
| / | Chr14 | 24839572 |  |  | <0.01 | 0.99 |
| CC |  |  | 130 | 69 |  |  |
| CG |  |  | 1 | 0 |  |  |
| / | Chr14 | 24839648 |  |  | <0.01 | 0.99 |
| CC |  |  | 130 | 69 |  |  |
| CG |  |  | 1 | 0 |  |  |
| rs2229310 | Chr14 | 24839756 |  |  | 0.01 | 0.99 |
| CC |  |  | 131 | 67 |  |  |
| CT |  |  | 0 | 2 |  |  |
| rs778422832 | Chr14 | 24839812 |  |  | <0.01 | 0.99 |
| GG |  |  | 130 | 69 |  |  |
| GA |  |  | 1 | 0 |  |  |
| rs56044944 | Chr14 | 24839897 |  |  | <0.01 | 0.99 |
| TT |  |  | 130 | 69 |  |  |
| TA |  |  | 1 | 0 |  |  |
| / | Chr14 | 24839930 |  |  | <0.01 | 0.99 |
| AA |  |  | 130 | 69 |  |  |
| AT |  |  | 1 | 0 |  |  |
| rs10141527 | Chr14 | 24841517 |  |  | 0.08 | 0.96 |
| AA |  |  | 3 | 1 |  |  |
| AG |  |  | 34 | 18 |  |  |
| GG |  |  | 94 | 50 |  |  |
| / | Chr14 | 24841650 |  |  | <0.01 | 0.99 |
| CC |  |  | 130 | 69 |  |  |
| CT |  |  | 1 | 0 |  |  |
| / | Chr14 | 24841698 |  |  | <0.01 | 0.99 |
| GG |  |  | 131 | 68 |  |  |
| GC |  |  | 0 | 1 |  |  |
| rs773652389 | Chr14 | 24842421 |  |  | <0.01 | 0.99 |
| CC |  |  | 130 | 69 |  |  |
| CT |  |  | 1 | 0 |  |  |
| rs149390527 | Chr14 | 24842498 |  |  | <0.01 | 0.99 |
| CC |  |  | 130 | 69 |  |  |
| CT |  |  | 1 | 0 |  |  |
| rs77893724 | Chr14 | 24842563 |  |  | 0.01 | 0.99 |
| AA |  |  | 131 | 67 |  |  |
| AC |  |  | 0 | 2 |  |  |
| rs762460806 | Chr14 | 24843537 |  |  | <0.01 | 0.99 |
| CC |  |  | 130 | 69 |  |  |
| CT |  |  | 1 | 0 |  |  |
| rs2295298 | Chr14 | 24843620 |  |  | 5.20 | 0.074 |
| TT |  |  | 101 | 50 |  |  |
| TC |  |  | 26 | 15 |  |  |
| CC |  |  | 4 | 4 |  |  |
| / | Chr14 | 24843697 |  |  | <0.01 | 0.99 |
| GG |  |  | 130 | 69 |  |  |
| GC |  |  | 1 | 0 |  |  |
| rs12890614 | Chr14 | 24843699 |  |  | 5.20 | 0.074 |
| CC |  |  | 101 | 50 |  |  |
| CT |  |  | 26 | 15 |  |  |
| TT |  |  | 4 | 4 |  |  |
| rs368284613 | Chr14 | 24843712 |  |  | <0.01 | 0.99 |
| CC |  |  | 130 | 69 |  |  |
| CG |  |  | 1 | 0 |  |  |
| rs12880769 | Chr14 | 24845154 |  |  | 5.20 | 0.074 |
| CC |  |  | 101 | 50 |  |  |
| CA |  |  | 26 | 15 |  |  |
| AA |  |  | 4 | 4 |  |  |
| rs56111443 | Chr14 | 24845338 |  |  | 0.11 | 0.95 |
| AA |  |  | 125 | 66 |  |  |
| AT |  |  | 6 | 3 |  |  |
| rs10141896 | Chr14 | 24845402 |  |  | 0.22 | 0.89 |
| GG |  |  | 70 | 36 |  |  |
| GT |  |  | 53 | 28 |  |  |
| TT |  |  | 8 | 5 |  |  |
| / | Chr14 | 24845678 |  |  | <0.01 | 0.99 |
| GG |  |  | 130 | 69 |  |  |
| GA |  |  | 1 | 0 |  |  |
| rs7149586 | Chr14 | 24845841 |  |  | 5.20 | 0.074 |
| TT |  |  | 101 | 50 |  |  |
| TC |  |  | 26 | 15 |  |  |
| CC |  |  | 4 | 4 |  |  |
| rs2243891 | Chr14 | 24846757 |  |  | 0.08 | 0.96 |
| AA |  |  | 3 | 1 |  |  |
| AG |  |  | 34 | 18 |  |  |
| GG |  |  | 94 | 50 |  |  |
| rs10362 | Chr14 | 24846961 |  |  | 0.35 | 0.84 |
| GG |  |  | 92 | 48 |  |  |
| GT |  |  | 35 | 21 |  |  |
| TT |  |  | 4 | 0 |  |  |
| rs56006071 | Chr14 | 24847063 |  |  | <0.01 | 0.99 |
| CC |  |  | 130 | 69 |  |  |
| CA |  |  | 1 | 0 |  |  |
| / | Chr14 | 24847105 |  |  | <0.01 | 0.99 |
| AA |  |  | 131 | 68 |  |  |
| AC |  |  | 0 | 1 |  |  |
