# Supplementary material for: Polymorphisms of nucleotide factor of activated T cells cytoplasmic 2 and 4 and the risk of acute rejection following kidney transplantation
Source: World J Urol. 2017 Nov 4;36(1):111–6. doi: 10.1007/s00345-017-2117-2 (PMC5758697; doi:10.1007/s00345-017-2117-2)
Supplement: Supplementary file 5 — Supplementary material 5 (DOCX 28 kb) [file 345_2017_2117_MOESM5_ESM.docx]

**(Supplemental) Table 5. Regression analysis for age-, gender- and immunosuppressive protocol-adjusted *NFATC2、NFATC4* genetic polymorphisms among recipients with acute rejection.**

| SNPs | model | OR | 95%CIs | *P* value |
| --- | --- | --- | --- | --- |
| rs139882911 |  |  |  |  |
|  | Additive | 1.56 | 0.51, 4.80 | 0.44 |
|  | Dominant | 1.56 | 0.51, 4.80 | 0.44 |
| rs228840 |  |  |  |  |
|  | Additive | 1.02 | 0.62, 1.68 | 0.95 |
|  | Dominant | 0.99 | 0.54, 1.85 | 0.98 |
|  | Recessive | 1.14 | 0.31, 4.15 | 0.84 |
|  | HOM | 1.13 | 0.31, 4.19 | 0.85 |
|  | HET | 0.97 | 0.51, 1.87 | 0.94 |
| rs73615391 |  |  |  |  |
|  | Additive | 2.22 | 0.12, 41.42 | 0.59 |
|  | Dominant | 2.22 | 0.12, 41.42 | 0.59 |
| rs56332276 |  |  |  |  |
|  | Additive | 0.87 | 0.48, 1.57 | 0.64 |
|  | Dominant | 0.83 | 0.41, 1.67 | 0.60 |
|  | Recessive | 0.93 | 1.56, 5.57 | 0.94 |
|  | HOM | 0.89 | 0.15, 5.35 | 0.90 |
|  | HET | 0.82 | 0.40, 1.71 | 0.60 |
| rs6013193 |  |  |  |  |
|  | Additive | 0.97 | 0.62, 1.51 | 0.88 |
|  | Dominant | 0.87 | 0.47, 1.61 | 0.65 |
|  | Recessive | 1.16 | 0.49, 2.73 | 0.73 |
|  | HET | 0.82 | 0.43, 1.58 | 0.56 |
|  | HOM | 1.04 | 0.41, 2.64 | 0.94 |
| rs12479626 |  |  |  |  |
|  | Additive | 0.85 | 0.36, 2.00 | 0.71 |
|  | Dominant | 0.85 | 0.36, 2.00 | 0.71 |
| rs6021231 |  |  |  |  |
|  | Additive | 1.01 | 0.65, 1.55 | 0.98 |
|  | Dominant | 0.89 | 0.48, 1.66 | 0.71 |
|  | Recessive | 1.24 | 0.56, 2.77 | 0.59 |
|  | HET | 0.82 | 0.42, 1.59 | 0.56 |
|  | HOM | 1.11 | 0.46, 2.69 | 0.82 |
| rs74644406 |  |  |  |  |
|  | Additive | 2.04 | 0.62, 6.73 | 0.24 |
|  | Dominant | 2.04 | 0.62, 6.73 | 0.24 |
| rs3746420 |  |  |  |  |
|  | Additive | 1.85 | 0.77, 4.39 | 0.17 |
|  | Dominant | 1.85 | 0.77, 4.39 | 0.17 |
| rs2869427 |  |  |  |  |
|  | Additive | 0.82 | 0.36, 1.89 | 0.65 |
|  | Dominant | 0.87 | 0.36, 2.08 | 0.76 |
|  | HET | 0.93 | 0.39, 2.24 | 0.88 |
| rs75374025 |  |  |  |  |
|  | Additive | 0.95 | 0.39, 2.03 | 0.91 |
|  | Dominant | 1.07 | 0.40, 2.87 | 0.88 |
|  | HET | 1.23 | 0.45, 3.39 | 0.68 |
| rs55651033 |  |  |  |  |
|  | Additive | 0.57 | 0.15, 2.23 | 0.42 |
|  | Dominant | 0.57 | 0.15, 2.23 | 0.42 |
| rs45565135 |  |  |  |  |
|  | Additive | 0.97 | 0.54,1.74 | 0.92 |
|  | Dominant | 1.13 | 0.58,2.16 | 0.72 |
|  | HET | 1.27 | 0.65,2.46 | 0.48 |
| rs537893669 |  |  |  |  |
|  | Additive | 1.88 | 0.12,30.94 | 0.66 |
|  | Dominant | 1.88 | 0.12,30.94 | 0.66 |
| rs1955915 |  |  |  |  |
|  | Additive | 1.09 | 0.71,1.69 | 0.67 |
|  | Dominant | 1.11 | 0.61,2.02 | 0.73 |
|  | Recessive | 1.18 | 0.46,2.99 | 0.73 |
|  | HET | 1.08 | 0.57,2.06 | 0.81 |
|  | HOM | 1.22 | 0.46,3.21 | 0.69 |
| rs2229309 |  |  |  |  |
|  | Additive | 1.29 | 0.75,2.24 | 0.35 |
|  | Dominant | 1.31 | 0.66,2.59 | 0.44 |
|  | Recessive | 1.83 | 0.43,7.75 | 0.41 |
|  | HET | 1.21 | 0.58,2.54 | 0.61 |
|  | HOM | 1.91 | 0.45,8.16 | 0.38 |
| rs2228233 |  |  |  |  |
|  | Additive | 0.98 | 0.65,1.47 | 0.92 |
|  | Dominant | 1.29 | 0.66,2.53 | 0.45 |
|  | Recessive | 0.69 | 0.33,1.43 | 0.32 |
|  | HET | 1.54 | 0.75,3.13 | 0.23 |
|  | HOM | 0.90 | 0.38,2.13 | 0.81 |
|  |  |  |  |  |
| rs10141527 |  |  |  |  |
|  | Additive | 0.87 | 0.48, 1.59 | 0.67 |
|  | Dominant | 0.91 | 0.47, 1.78 | 0.79 |
|  | Recessive | 0.48 | 0.047, 4.77 | 0.53 |
|  | HET | 0.96 | 0.48, 1.9 | 0.90 |
|  | HOM | 0.47 | 0.046, 4.75 | 0.52 |
| rs2295298 |  |  |  |  |
|  | Additive | 1.27 | 0.74, 2.18 | 0.39 |
|  | Dominant | 1.27 | 0.64, 2.51 | 0.49 |
|  | Recessive | 1.83 | 0.43, 7.75 | 0.41 |
|  | HET | 1.17 | 0.56, 2.43 | 0.68 |
|  | HOM | 1.89 | 0.44, 8.11 | 0.39 |
| rs12890614 |  |  |  |  |
|  | Additive | 1.27 | 0.74, 2.19 | 0.39 |
|  | Dominant | 1.27 | 0.64, 2.50 | 0.49 |
|  | Recessive | 1.83 | 0.43, 7.75 | 0.41 |
|  | HET | 1.17 | 0.56, 2.43 | 0.68 |
|  | HOM | 1.89 | 0.44, 8.11 | 0.38 |
| rs12880769 |  |  |  |  |
|  | Additive | 1.27 | 0.74, 2.18 | 0.39 |
|  | Dominant | 1.27 | 0.64, 2.51 | 0.49 |
|  | Recessive | 1.83 | 0.43, 7.75 | 0.41 |
|  | HET | 1.17 | 0.56, 2.43 | 0.68 |
|  | HOM | 1.89 | 0.44, 8.11 | 0.38 |
| rs56111443 |  |  |  |  |
|  | Additive | 0.95 | 0.22, 4.05 | 0.95 |
|  | Dominant | 0.95 | 0.22, 4.05 | 0.95 |
| rs10141896 |  |  |  |  |
|  | Additive | 1.09 | 0.68, 1.77 | 0.71 |
|  | Dominant | 1.08 | 0.59, 1.97 | 0.79 |
|  | Recessive | 1.25 | 0.38, 4.08 | 0.71 |
|  | HET | 1.05 | 0.56, 1.97 | 0.86 |
|  | HOM | 1.28 | 0.38, 4.31 | 0.68 |
| rs7149586 |  |  |  |  |
|  | Additive | 1.27 | 0.74, 2.18 | 0.39 |
|  | Dominant | 1.27 | 0.64, 2.51 | 0.49 |
|  | Recessive | 1.83 | 0.43, 7.75 | 0.41 |
|  | HET | 1.17 | 0.56, 2.43 | 0.68 |
|  | HOM | 1.89 | 0.44, 8.11 | 0.39 |
| rs2243891 |  |  |  |  |
|  | Additive | 0.87 | 0.48, 1.59 | 0.67 |
|  | Dominant | 0.91 | 0.47, 1.78 | 0.79 |
|  | Recessive | 0.47 | 0.047, 4.77 | 0.53 |
|  | HET | 0.96 | 0.48, 1.9 | 0.90 |
|  | HOM | 0.47 | 0.046, 4.75 | 0.52 |
| rs10362 |  |  |  |  |
|  | Additive | 1.01 | 0.56, 1.84 | 0.96 |
|  | Dominant | 1.16 | 0.6, 2.23 | 0.65 |
|  | HET | 1.28 | 0.66, 2.50 | 0.45 |

Abbreviations: SNPs, single nuclotide polymorphisms; OR, odds ratio; CIs: confidential intervals.
